# Retraction: *In situ* fabrication of lanthanum-doped nickel oxide nanostructures using sol–gel for the degradation of rhodamine B

**DOI:** 10.1039/d5ra90016f

**Published:** 2025-02-20

**Authors:** Furqan Ali, Asma Nazir, Zeshan Ali Sandhu, Arslan Mehmood, Muhammad Asam Raza, Muhammad Hamayun, Abdullah G. Al-Sehemi

**Affiliations:** a Department of Physics, Faculty of Science, University of Sialkot Sialkot 51310 Pakistan; b Department of Chemistry, Faculty of Science, University of Gujrat Hafiz Hayat Campus Gujrat 50700 Pakistan zeshansandhu89@gmail.com asamgcu@yahoo.com; c Research Center for Advanced Materials Science (RCAMS), King Khalid University Abha 61413 Saudi Arabia; d Department of Chemistry, College of Science, King Khalid University Abha 61413 Saudi Arabia

## Abstract

Retraction of ‘*In situ* fabrication of lanthanum-doped nickel oxide nanostructures using sol–gel for the degradation of rhodamine B’ by Furqan Ali *et al.*, *RSC Adv.*, 2024, **14**, 4406–4415, https://doi.org/10.1039/D3RA08311J.

The Royal Society of Chemistry hereby wholly retracts this *RSC Advances* article due to concerns with the reliability of the data.

There are duplications within the SEM images in Fig. 4 (panels a & c and b & d), there are concerns with the labelling of the EDS spectra in Fig. 5, the XRD patterns in Fig. 6 have unusual similarity between the traces for 3% and 2% La doped NiO, and the UV-vis absorbance spectra in Fig. 7a have unusual peak shapes. The authors have admitted a mistake in the EDS and requested a correction for Fig. 4 and 5 but the raw data supplied by the authors for Fig. 6 and 7 show signs of manipulation.

Given the significance of these concerns, the Editor has lost confidence that the findings presented in this paper are reliable.

The authors were informed but have not indicated whether they agree with the decision to retract.

Signed: Laura Fisher, Executive Editor, *RSC Advances*

Date: 11th February 2025

